# Supplementary material for: Suspicious Positive Peritoneal Cytology (Class III) in Endometrial Cancer Does Not Affect Prognosis
Source: J Clin Med. 2022 Nov 3;11(21):6527. doi: 10.3390/jcm11216527 (PMC9657754; doi:10.3390/jcm11216527)
Supplement: Supplementary file 1 [file jcm-11-06527-s001.zip › Table S1.pdf]

§ Supplementary Table S1: Comparisons of clinicopathological characteristics according peritoneal cytological diagnosis

	Negative (n = 553)	Suspicious (n = 39)	Positive (n = 78)	p-values
FIGO stage, n (%)				< 0.001
I/II	471 (85.2)	27 (69.2)	38 (48.7)	
III/IV	82 (14.8)	12 (30.8)	40 (51.3)	
Myometrial invasion, n (%)				0.0295
< 1/2	382 (69.1)	27 (69.2)	42 (53.8)	
≥ 1/2	171 (30.9)	12 (30.8)	36 (46.2)	
LN metastasis, n (%)				0.00124
Positive	61 (11.0)	9 (23.1)	22 (28.2)	
Negative	492 (89.0)	30 (76.9)	56 (71.8)	
Histology, n (%)				< 0.001
EMG1/G2	457 (82.6)	35 (89.7)	49 (62.8)	
EMG3/Others	96 (17.4)	4 (10.3)	29 (37.2)	
Distant metastasis, n (%)				< 0.001
Positive	7 (1.3)	0 (0)	11 (14.1)	
Negative	546 (98.7)	39 (100)	67 (85.9)	
Adnexal metastasis, n (%)				0.0061
Positive	14 (2.5)	3 (7.7)	7 (9.0)	
Negative	539 (97.5)	36 (92.3)	71 (91.0)	

§, Kruskal-Wallis test; other statistical analyses were conducted by Fisher’s test

LN, lymph node; EMG1/2/3, endometrioid carcinoma grade 1/2/3
