# Supplementary material for: Suspicious Positive Peritoneal Cytology (Class III) in Endometrial Cancer Does Not Affect Prognosis
Source: J Clin Med. 2022 Nov 3;11(21):6527. doi: 10.3390/jcm11216527 (PMC9657754; doi:10.3390/jcm11216527)
Supplement: Supplementary file 1 [file jcm-11-06527-s001.zip › Table S2.pdf]

Supplementary Table S2 Clinical information of peritoneal suspicious cytology

Case	Age	Stage	pT	Lymph node metastasis	Omental metastasis	Adnexal metastasis	Histological type	Omentectomy
1	36	1A	1A	−	−	−	G1	+
2	40	IA	1a	−	−	−	G1	+
3	42	II	2	−	−	−	G1	+
4	44	II	2	−	−	−	G1	−
5	44	IA	1a	−	−	−	G1	+
6	47	IA	1a	−	−	−	G1	+
7	47	IIIA	3a	−	−	+	G1	+
8	47	IIIA	3a	−	−	+	G2	−
9	48	IA	1a	−	−	−	G1	−
10	50	IA	1a	−	−	−	G3	+
11	51	IA	1a	−	−	−	G1	−
12	53	IA	1a	−	−	−	G1	−
13	53	IA	1a	−	−	−	G1	−
14	55	IA	1a	−	−	−	G2	+
15	58	IB	1b	−	−	−	G3	+
16	59	IB	1b	−	−	−	G2	−
17	62	IB	1b	−	−	−	G1	+
18	55	IA	1a	−	−	−	G1	−

19	74	IA	1a	−	−	−	G3	−
20	53	IIIC 2	2	+	−	−	G1	+
21	55	IIIC2	2	+	−	−	G2	−
22	53	3C2	2	+	−	−	G2	+
23	51	1A	1a	−	−	−	G1	−
24	54	1A	1a	−	−	−	G1	−
25	44	1A	1a	−	−	−	G1	+
26	41	1A	1a	−	−	−	G1	+
27	51	1A	1a	−	−	−	G3	+
28	33	1A	1a	−	−	−	G1	−
29	40	3C2	1b	+	−	−	G1	−
30	51	3C2	1b	+	−	−	G2	+
31	53	3C1	1b	+	−	−	G2	−
32	56	3C1	1b	+	−	−	G2	−
33	41	1A	1a	−	−	−	G1	−
34	70	3C2	3a	+	−	−	G2	−
35	42	1A	1a	−	−	−	G1	−
36	56	1A	1a	−	−	−	G1	−
37	46	3A	3a	−	−	+	G1	−
38	50	1A	1a	−	−	−	G1	−
39	52	3C2	2	+	−	−	G1	−
